# Stress Hormones: Unveiling the Role in Accelerated Cellular Senescence

**DOI:** 10.14336/AD.2024.0262

**Published:** 2025-07-16

**Authors:** Tian Qin, Tao Chen, Rui Ma, Huan Li, Cui Li, Jin Zhao, Jinguo Yuan, Zuoming Zhang, Xiaoxuan Ning

**Affiliations:** ^1^Department of Geriatrics, Xijing Hospital, Fourth Military Medical University, Xi'an, China.; ^2^Department of Clinical Aerospace Medicine, Fourth Military Medical University, Xi’an, China.; ^3^Department of Nephrology, Xijing Hospital, Fourth Military Medical University, Xi'an, China.

**Keywords:** cellular senescence, stress-induced premature senescence, stress hormones, oxidative stress, DNA damage

## Abstract

Cellular senescence is a complex process involving multiple factors, such as genetics, environment, and behavior. However, recent studies have shown that stress also plays a crucial role in inducing cellular senescence. Stress can affect cellular function and structure through various pathways, leading to accelerated aging. Exposure to stressful conditions can alter the neuroendocrine system, activate the hypothalamus-pituitary-adrenal axis and sympathetic adrenal medullary axis, and release cortisol and catecholamines, causing mitochondrial dysfunction, generating excessive reactive oxygen species, and inducing oxidative stress, DNA damage, and inflammatory reactions, ultimately resulting in accelerated cellular senescence. The process of stress-induced cellular senescence has been implicated in a number of chronic diseases, including age-related macular degeneration, chronic kidney disease, type 2 diabetes, cardiovascular disease and obstructive sleep apnea. In this review, we integrate recent progress research progress in our understanding of the mechanisms of stress-induced cellular senescence and discuss its underlying mechanisms from the perspective of stress hormones. We review potential therapeutic targets for stress-induced premature senescence and discuss the advantages and limitations of existing pharmacological agents capable of ameliorating stress-induced premature senescence.

## 1. Introduction

Cellular senescence is characterized by a durable cessation of the cell cycle accompanied by predetermined phenotypic alterations, as initially observed by Hayflick and Moorhead in 1961 when human diploid cells exhibited an irreversible growth arrest following a specific number of divisions in vitro [1, 2]. This phenomenon is known as replicative senescence, and the limit of cellular proliferation as the Hayflick limit [3]. This discovery led to cellular senescence becoming a research hotspot in the biomedical field. Subsequently, scholars reported that exposure of cells to H_2_O_2_, hyperoxia, or tert-butylhydroperoxide could induce cellular senescence in human keratinocytes, fibroblasts, melanocytes and umbilical vascular endothelial cells [4]. This phenomenon, in which cells exposed to subcytotoxic stress conditions undergo irreversible growth arrest, is referred to as stress-induced premature senescence (SIPS) [5]. Furthermore, researchers detected cellular senescence during the initial days of organismal development (embryonic stage), a process referred to as developmentally programmed senescence (DPS) [6]. These represent the three identified pathways of cellular senescence. Their similarities and differences are summarized in Table 1.

Stress manifests as a crucial adaptive physiological response to the environment, as well as a maladaptive, non-specific dysregulation of this physiological response [7]. Stress is a pervasive factor throughout human life. A cross-sectional study focusing on women showed a correlation between high perceived stress levels and shorter telomere length, suggesting a negative impact of stress on telomeres [8]. Another randomized controlled trial found that patients with stress, adjustment disorders, depression, and anxiety had shorter leukocyte telomere length compared to healthy individuals [9]. A systematic review suggested a potential association between chronic psychological stress disorders such as post-traumatic stress disorder, anxiety, and depression and telomere shortening [10]. These research findings have significantly enhanced our comprehension of the influence of stress on cellular aging. Given the close association between cellular aging and the occurrence and progression of various diseases, these findings could translate into the prevention and treatment of related conditions in the field of medicine, although there is currently limited research analyzing stress hormone-induced cellular senescence. This review aims to integrate recent studies and discuss the mechanisms of stress hormone-induced cellular senescence.

**Table 1:** A summary of the differences between replicative senescence (RS), stress-induced premature senescence (SIPS), and developmentally programmed senescence (DPS).

	RS	SIPS	DPS
**Condition of occurrence**	Cells that proliferate to the Hayflick limit [3]	It is induced by various stressors, such as ultraviolet radiation, oxidative stress, and oncogene activity [219]	Occurs at the embryonic stage [6]
**Telomeres**	Telomeres shortening [57]	Telomeres shortened but not to the same extent as RS [220]	Telomere shortening [221]
**DNA damage response**	When telomeres shorten to the Hayflick limit, the telomere ends become exposed, triggering a classic DNA damage repair response and leading the cell cycle into a state of senescent arrest [57]	Caused by the accumulation of reactive oxygen species and other substances and is the main cause of cellular aging [67]	No signs of DNA damage are detected in developmentally aging cells, nor is there evidence of activation of DNA damage-dependent kinases such as ATM and ATR [221]

ATM: ataxia telangiectasia mutated; ATR: ataxia telangiectasia and Rad3-related

## 2. Mechanism of Stress Hormone-induced Premature Senescence

During periods of stress, the neuroendocrine system responds with the activation of the sympathetic-adrenal medulla (SAM) system and the hypothalamus-pituitary-adrenal (HPA) axis representing the most important neuroendocrine responses. During stressful situations, the paraventricular nucleus releases corticotropin-releasing hormone and the anterior pituitary gland releases adrenocorticotropic hormone (ACTH) into the bloodstream. ACTH stimulates the release of cortisol from the adrenal glands [11]. SAM also responses to stress by increasing catecholamine levels, including adrenaline and noradrenaline [12]. These interactions induce specific changes in the body to adapt to external environmental changes. The key characteristic of SIPS is DNA damage, caused by changes in stress hormone levels that increase oxidative stress.

## 2.1 Basics of telomere biology

Telomeres are specialized DNA-protein complexes located at the ends of chromosomes. Comprising short repetitive DNA sequences bound by telomere-binding proteins, these structures form unique "cap" formations. Their primary function is to maintain chromosomal integrity and regulate the cell division cycle [13, 14]. DNA replication leads to telomere shortening, triggering a DNA damage response (DDR) when telomeres reach a critical length. Concurrently, these shortened telomeres retain sufficient telomere-binding proteins, thereby eliciting sustained DDR and inducing cell cycle arrest [14]. The length of telomeres is regulated by telomerase, primarily composed of telomerase reverse transcriptase and telomerase RNA. Telomerase is a DNA polymerase that continuously synthesizes multiple telomeric repeat sequences to elongate the 3' end of chromosomes, counteracting telomere attrition. Therefore, telomerase plays a pivotal role in maintaining telomere stability and functionality [15].

Telomeres are covered by a specialized protein complex known as the shelterin complex, which consists of six protein subunits: TRF1, TRF2, TPP1, POT1, TIN2, and RAP. This complex prevents the occurrence of DDR while simultaneously regulating the activity of telomerase [13, 16]. Mutations in the aforementioned protein subunits disrupt the structure of the shelterin complex, leading to telomere shortening. In vitro studies have shown that degradation of TRF1 and TRF2 significantly shortens telomeres [17]. In summary, telomere shortening, telomerase deficiency, and disruption of the shelterin complex structure all contribute to cellular senescence.

Currently, methods for measuring telomeres include quantitative polymerase chain reaction, terminal restriction fragment analysis (southern blot), fluorescence in situ hybridization based techniques, telomere dysfunctional induced foci analysis, telomere shortest length assay, single telomere length analysis methods, single-cell telomere length measurement pqPCR, single telomere absolute-length rapid assay, optical mapping in nano-channel array, telomere length combing assay, and sequencing based estimation techniques [18, 19]. There is substantial evidence indicating that the shortest telomeres trigger DNA damage responses, thereby contributing to replicative senescence in mammals [18]. The length of the shortest telomeres is a critical biomarker determining cellular fate and the onset of aging. However, current methods for detecting short telomeres are time-consuming, labor-intensive, and fail to meet high throughput requirements [18]. Therefore, methods for measuring telomere length are not superior or inferior; each has its own advantages. The telomere length of peripheral blood mononuclear cells has been proposed as a surrogate marker for telomere length in the entire organism. Due to the invasive nature of telomere length analysis in solid tissues requiring biopsy, telomere length in peripheral blood leukocytes has been suggested as an alternative marker for telomere length in other tissues [20]. However, some scholars question the practicality of using telomere length in peripheral blood mononuclear cells as a biomarker for individual aging assessments [20]. This study found significant variations in relative telomere lengths among various organs in rats. The longest telomeres are found in skeletal muscle, while the shortest telomeres are found in colonic tissue, with a difference in length of approximately twofold. In human research, similar findings have been reported. Demanelis et al. [21] observed that telomeres are shortest in human leukocytes, while the longest telomeres are found in testes, colon, and skeletal muscle, with differences of up to 2.5-fold. Therefore, further research is needed to validate whether the telomere length of peripheral blood mononuclear cells can represent telomere function across the entire organism.

## 2.2 Hypothalamus-pituitary-adrenal (HPA) axis

The HPA axis plays a crucial role in stress responses. In this section, we will focus on discussing how glucocorticoid contributes to SIPS. Animal experiments have shown that chronic stress in rats and long-term corticosterone treatment in mice significantly shorten the length of the dentate gyrus and genomic DNA telomeres in blood [22]. In addition, adult mouse bone marrow adipocyte lineage cells accelerated cell aging after long-term glucocorticoid treatment [23]. In vitro experiments using dexamethasone treatment on lung adenocarcinoma and mouse hippocampal cell lines revealed irreversible cellular senescence in the former and significant telomere shortening in the latter [22, 24]. These experimental results suggest that long-term exposure to chronic stress can lead to cellular senescence.

## 2.2.1 Glucocorticoids induce mitochondrial dysfunction

The HPA axis is activated when the organism is under stress, which leads to the release of cortisol. Glucocorticoid is a hormone that plays a major role in stress response. Glucocorticoid first binds to the glucocorticoid receptor (GR), which is an important member of the nuclear receptor superfamily that controls a variety of physiological functions, such as, metabolism, development, and reproduction [25, 26]. GR exerts its influence ubiquitously across diverse bodily tissues and cellular structures, and includes almost all cells as its target [27]. Like other nuclear receptors, GRs function as ligand-activated transcription factors, translocating from the cytoplasm to the nucleus, where they interact with glucocorticoid response elements in the genome to modulate cellular function [28].

Glucocorticoid governs a plethora of physiological mechanisms necessitating heightened energy outlay, with the modulation of mitochondrial energy metabolism emerging as a primary facet of their regulatory repertoire [29]. Mitochondria are acknowledged as pivotal constituents of the stress response owing to their role in energy provision and their capacity to orchestrate signaling cascades conducive to stress adaptation [29, 30]. In vitro studies have shown the presence of GRs in mitochondria from different tissues and cell types [31]. Glucocorticoid response elements are also found in mitochondrial DNA (mtDNA), suggesting a potential role in regulating mtDNA transcription [32]. Glucocorticoid modulates mtDNA transcription by specifically binding to glucocorticoid response elements in the D-loop region of mtDNA via GR [28, 32]. This indicates that glucocorticoid is closely related to mitochondrial function.

The physiological response to glucocorticoids follows a biphasic, inverted U-shape dose-response curve [33]. The affinity of GR binding to mtDNA peaks at a dosage of 0.3 mg/kg, yet diminishes at a dosage of 3 mg/kg [34]. Although short-term exposure to glucocorticoid is considered a defensive mechanism linked with the stimulation of mitochondrial biogenesis and increased activity of respiratory chain enzymes, prolonged glucocorticoid exposure has been shown to decrease mitochondrial membrane potential, suppress mitochondrial biogenesis, impair ATP production, leading to excessive generation of reactive oxygen species (ROS), ultimately resulting in mitochondrial dysfunction [29, 30, 35].

Chronic excessive glucocorticoid can directly impair mitochondrial function. Physiological concentrations of glucocorticoid can elevate the expression of genes within both the mitochondrial and nuclear genomes, augment mitochondrial membrane potential, mitigate programmed cell death, fulfill elevated cellular energy requisites, foster the genesis of new mitochondria, and increase mitochondrial DNA content, thereby enhancing the capacity for energy production by mitochondria [36]. However, prolonged exposure to high doses of glucocorticoid can instead impede electron transport within the mitochondrial respiratory chain, diminish mitochondrial membrane potential, and culminate in a diminished capacity for ATP synthesis [30]. Nuclear GR elevates the expression of bcl-2-associated X protein, which in turn destabilizes the outer mitochondrial membrane and diminishes mitochondrial membrane potential. The alterations in mitochondrial membrane potential induced by corticosteroids can precipitate the liberation of cytochrome c from mitochondria into the cytoplasm, thus perturbing mitochondrial equilibrium [37]. The same findings have been observed in animal experiments, glucocorticoid directly inhibits cytochrome c oxidase activity in rat kidneys mitochondria and suppresses the activities of mitochondrial complex I and V in rat brains, leading to a decrease in respiratory control ratio [38, 39]. Dysfunction within the mitochondrial respiratory chain precipitates an overabundance of ROS, thereby instigating oxidative stress. These experimental findings indicate that prolonged exposure to high levels of glucocorticoids can disrupt mitochondrial function through multiple targets, leading to mitochondrial dysfunction.

**Figure 1.**
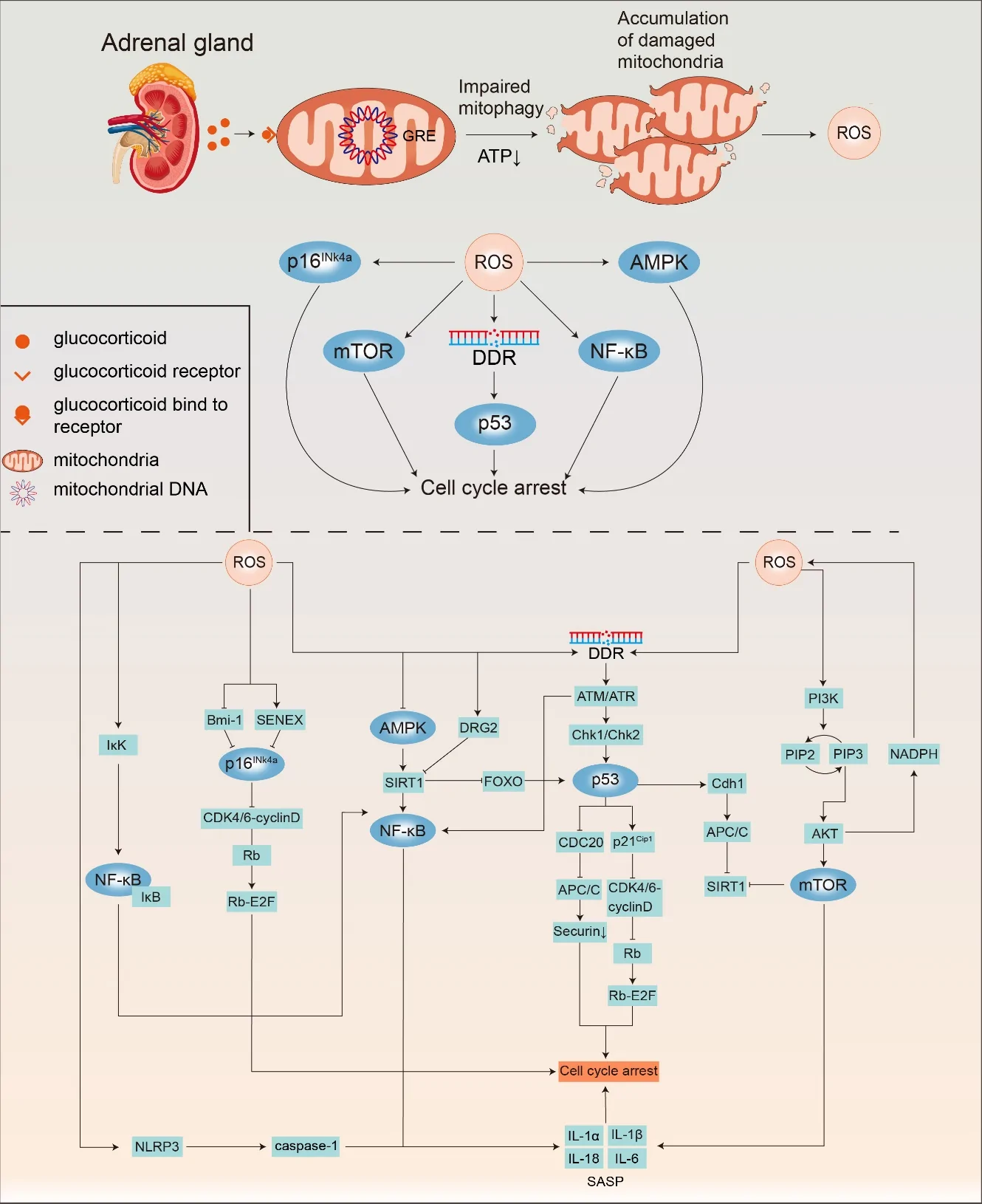
**Mechanisms underlying cellular senescence induced by glucocorticoids**. Stress activates the hypothalamic-pituitary-adrenal axis, leading to abnormal secretion of glucocorticoids. Glucocorticoids exert their effects through glucocorticoid receptors and interact with glucocorticoid response elements in mitochondrial DNA. On one hand, long-term high-dose glucocorticoid administration causes a decrease in mitochondrial ATP synthesis capacity, resulting in excessive generation of reactive oxygen species. On the other hand, glucocorticoids inhibit mitochondrial autophagy, leading to the accumulation of damaged mitochondria and further increasing ROS production. ROS primarily induce cell cycle arrest through the activation of the p16, mTOR, p53, NF-κB, and AMPK signaling pathways. AKT: protein kinase B; AMPK: adenosine monophosphate-activated protein kinase; APC/C: anaphase-promoting complex/cyclosome; ATM: ataxia telangiectasia mutated; ATR: ataxia telangiectasia and Rad3-related; Bmi-1: B-cell-specific Moloney murine leukemia virus integration site 1; CDC20: cell division cycle protein 20 homolog; CDK: cyclin-dependent kinase; Chk: checkpoint kinase; DDR: DNA damage response; DRG2: developmentally regulated GTP-binding protein 2; FOXO: forkhead box O; GRE: glucocorticoid response elements; IL: interleukin; IκB: NF-κB inhibitor; IκK: NF-κB inhibitor kinase; mTOR: mammalian target of rapamycin; NADPH: nicotinamide adenine dinucleotide phosphate; NF-κB: nuclear factor kappa-B; NLRP3: nucleotide-binding oligomerization domain, leucine rich repeat and pyrin domain-containing protein 3; PI3K: phosphoinositide 3-kinase; PIP2: phosphatidylinositol 4,5-bisphosphate; PIP3: phosphatidylinositol 3,4,5-trisphosphate; Rb: retinoblastoma protein; Rb-E2F: retinoblastoma protein-E2F transcription factor; ROS: reactive oxygen species; SASP: senescence-associated secretory phenotype; SIRT1: sirtuin

Glucocorticoid can additionally promote ROS generation by inhibiting mitochondrial autophagy and biogenesis. In vitro experiments have shown that glucocorticoid can directly guide the GR to bind to the peroxisome proliferator-activated receptor gamma coactivator 1-Alpha (PGC-1α) promoter, resulting in downregulated expression and nuclear translocation of PGC-1α, a critical regulator of mitochondrial biogenesis and autophagy [40]. This leads to inhibition of PGC-1α and its downstream targets, causing oxidative phosphorylation disorder, mitochondrial biogenesis impairment, reduced autophagic clearance, increased mitochondrial ROS, decreased ATP generation, and diminished gluconeogenesis [41, 42]. Furthermore, long-term high-dose glucocorticoid exposure can downregulate the expression of BCL2/adenovirus E1B 19 kDa protein-interacting protein 3-like (NIX), leading to impaired mitochondrial respiration and inhibition of NIX-mediated mitochondrial autophagy, independent of the parkin pathway [40]. In vitro, mitofusin 1/2 and SOD1/2, which play key roles in mitochondrial autophagy, are suppressed upon exposure to cortisol and stress, resulting in mitochondrial stasis and a reduced propensity for mitochondrial autophagy [43]. Finally, protein balance disorder caused by mitochondrial dysfunction can initiate SIPS; treatment with protease inhibitors can cause DNA damage and SIPS in human fibroblasts due to mitochondrial dysfunction and accumulation, resulting in excessive ROS production [44]. Impeding mitochondrial autophagy results in the buildup of impaired mitochondria, fostering heightened production of mitochondrial ROS, consequently exacerbating cellular damage. (Fig. 1).

## 2.2.2 Glucocorticoids induce the oxidative stress

The human body aims to maintain a balance between oxidation and antioxidation. Oxidative stress occurs if this balance is disrupted. At low concentrations, ROS assumes a pivotal role in maintaining cellular homeostasis. However, an abundance of ROS precipitates cellular dysfunction, instigates protein and lipid peroxidation, and incites DNA damage, culminating inexorably in irreversible cellular harm and cell death [45]. To maintain homeostasis, mammalian cells utilize a complex antioxidant defense system to eliminate ROS, converting them into non-toxic forms, which includes enzymes such as glutathione peroxidase, catalase, and superoxide dismutase (SOD) [46]. Mitochondria, the chief orchestrators of ATP synthesis via the electron transport chain in eukaryotic cells, additionally serve as the principal generators of ROS within cellular confines [47].

Nuclear factor kappa-B (NF-κB) is a major signaling factor in response to ROS, and is comprised of multiple subunits capable of swiftly triggering the transcriptional activation of genes implicated in immune inflammatory, and acute phase responses [48]. In the context of oxidative stress, ROS can activate NF-κB, leading to the activation of NF-κB inhibitor kinase [49], which phosphorylates NF-κB inhibitor, leading to its ubiquitin-dependent degradation. Free NF-κB translocates into the nucleus and binds to genes containing NF-κB binding sites, triggering transcription [49-51]. Nuclear factor-E2-related factor 2 (Nrf2) is the main antioxidant factor. Nrf2 belongs to a small family of transcription factors inducing a set of antioxidants and detoxication enzymes [52]. Nrf2 interacts with the antioxidant response element to uphold redox homeostasis [53]. In the homeostatic state, Nrf2 is ubiquitinated by Kelch-like ECH-associated protein 1 (Keap1) and exists in a complex form in the cytoplasm, remaining inactive. However, under oxidative stress, conformational changes occur in Keap1, resulting in dissociation of Nrf2 from Keap1 [53, 54]. As a result, Nrf2 binds to antioxidant response element and controls the expression of genes involved in antioxidant and anti-inflammatory responses, which reduces ROS levels and oxidative stress through the REGγ-GSK-3β-Nrf2 pathway [55]. Clearly, the activation of Nrf2/Keap1 exhibits antioxidant properties. However, Nrf2 functionality diminishes with advancing age, thereby heightening susceptibility to oxidative stress [56]. NF-κB and Nrf2 are regulatory factors involved in the redox balance, and stress-induced ROS increase is mainly regulated through these two pathways. Once ROS levels exceed the capacity of the organism to handle, it can lead to DNA damage.

### 2.2.3 Glucocorticoids induce the DNA damage

Telomeres undergo progressive shortening with each successive cell division in replicative senescence. When the proliferative capacity of cells reaches the Hayflick limit, telomeres no longer shorten and DDR is activated [57]. In SIPS, cellular aging primarily arises from DDR triggered by the accrual of ROS and other bioactive compounds. Upon the onset of DDR, pivotal DNA damage sensing elements such as ataxia telangiectasia mutated and ataxia telangiectasia and Rad3-related, along with subsequent downstream effectors including checkpoint kinase 1 and 2, become activated. These activated components then initiate the activation of the p53/p21^Cip1^ pathway, ultimately culminating in the induction of cell cycle arrest [58]. In the event of sustained DNA damage, these components cause prolonged activation of DDR signaling pathways, resulting in cellular senescence and an extended proliferation arrest [59]. ROS triggers DDR to activate the p53/p21^Cip1^ pathway. P21^Cip1^ interacts with cyclin-dependent kinase (CDK)2 and CDK4/6, thereby impeding the functionality of cyclin-CDK complexes. This action induces the dephosphorylation of retinoblastoma protein (Rb) and facilitates the assembly of the retinoblastoma protein-E2F transcription factor (Rb-E2F) complex [60, 61]. The formation of the Rb-E2F complex exerts inhibitory control over the transcriptional activity of numerous genes crucial for the progression through the G1/S transition phase of the cell cycle. Consequently, this regulatory mechanism prompts cell cycle arrest specifically at the G1 and S phases [62]. Recent in vitro research elucidated the role of a novel signaling molecule, cell division cycle protein 20 homolog (CDC20), in the mechanism of SIPS, thereby enhancing our understanding of the p53 signaling pathway. Stress-induced activation of the p53 pathway in cells led to down-regulation of CDC20. Loss of CDC20 promoted inhibition of the anaphase-promoting complex/cyclosome complex, leading to down-regulation of securin, which in combination with an unclear signaling mediator, promotes premature aging [63]. Further research is warranted to elucidate the nature of ambiguous signaling mediators.

Some scholars have found that the activation of p53 further exacerbates mitochondrial dysfunction. As DNA damage increases, cytoplasmic p53 levels rise, leading to the degradation of newly formed mitochondria while dysfunctional mitochondria maintain fusion and evade degradation. Additionally, activation of the mitochondrial enzyme monoamine oxidase A inhibits the expression of the mitochondrial degradation signal protein parkin, resulting in an increase in the quantity of dysfunctional mitochondria [64, 65]. This leads to the excessive production of ROS described earlier, further activating p53 and resulting in a malignant cycle. While the p53 pathway plays a key role in the occurrence of SIPS, further research is needed to understand this signaling pathway and its interplay with other signaling pathways. Gaining greater understanding of these interactions could provide a more profound theoretical foundation for preventing and treating senescence-related diseases.

## 2.2.3.1 ROS activates the p16-Rb signaling pathway

P16 is regulated by its upstream regulatory factor, B-cell-specific Moloney murine leukemia virus integration site 1 (Bmi-1), which is a transcriptional repressor and polycomb group protein involved in cell cycle regulation [66]. Bmi-1 maintains self-renewal and cell cycle progression by inhibiting the expression of p16 [66]. ROS inhibit the expression of Bmi-1, leading to the activation of p16^INK4a^ [67]. P16^INK4a^ binds to CDK4/6, inhibiting the formation of CDK4/6-cyclin D complexes and slowing down the cell cycle by preventing the phosphorylation of Rb [67]. In SIPS, the p16^INK4a^ pathway is regulated by the SENEX gene, which can activate p16^INK4a^ and Rb protein in endothelial cells under H_2_O_2_-mediated stress conditions [68]. SENEX triggers the activation of the p16/Rb pathway by upregulating both p16 mRNA and protein levels, concurrently activating Rb as a downstream effector, ultimately inducing cell cycle arrest [68]. In epithelial cells, it does not induce the formation of replicative senescence, suggesting a unique role for SENEX in SIPS [68]. However, our understanding of the precise role of SENEX in the mechanisms underlying SIPS remains limited and further research is needed to confirm its function in SIPS and elucidate its’ signaling pathways.

After the activation of p53 and p16, the expression of adenosine monophosphate-activated protein kinase (AMPK) pathway is inhibited. AMPK, as a cellular energy sensor, facilitates ATP generation and inhibits energy-consuming processes to maintain energy homeostasis [69]. AMPK exerts its anti-aging effects through nicotinamide adenine dinucleotide (NAD^+^) synthesis and inhibition of the NF-κB, signal transducer and activator of transcription 3 (STAT3), and mTOR pathways [70]. AMPK activates SIRT1, which in turn activates downstream PGC-1, forkhead box O1 (FOXO1), and FOXO3, intervening in the aging process [71].

## 2.2.3.2 The role of SIRT1 in cellular senescence

SIRT1 is a member of the sirtuin family, functioning as a highly conserved NAD^+^-dependent deacetylase, and playing a pivotal role in age-related diseases and cellular senescence [72, 73]. In mammals, seven members of the SIRT family (SIRT1 to SIRT7) have been identified [73], with SIRT1 the most widely studied. SIRT1 is ubiquitously expressed on human cells and is primarily localized within the nucleus and cytoplasm [73]. The activity of SIRT1 is regulated by levels of NAD^+^, and it is thus closely associated with cellular energy status. SIRT1 participates in regulating numerous critical cellular biological processes, including cellular senescence [74], autophagy [75], apoptosis [76], inflammatory responses [72] and metabolic regulation [77]. Moreover, SIRT1 is also important in the occurrence and progression of metabolic diseases such as dyslipidemia and diabetes [78] as well as neurodegenerative diseases such as Parkinson's disease and Alzheimer's disease [79].

In reaction to DNA damage and oxidative stress, the expression of SIRT1 protein is downregulated facilitated by E3 ligase (both MDM2 and CHFR)-mediated polyubiquitination, followed by proteasomal degradation [80]. During aging and various SIPS processes, the expression of SIRT1 protein undergoes a decline through lysosome-mediated autophagy-associated degradation [74]. Decreased SIRT1 expression activates downstream FOXO, which in turn activates p53, forming a vicious cycle [67]. FOXO genes are closely related to human longevity. Protein kinase B (AKT)-mediated phosphorylation of FOXO1 not only governs cell cycle arrest and resistance to oxidative stress via transcriptional control of self-renewal and stem cell maintenance but also triggers apoptosis through its pro-apoptotic capacity [81-83]. In SIPS, SIRT1 is degraded by Cdh1, by activator of the multi-subunit E3 ubiquitin ligase anaphase-promoting complex/cyclosome. Cdh1 is activated by p53 [80]. Furthermore, in vitro research has found that the ectopic expression of developmentally regulated guanosine triphosphate (GTP)-binding protein 2 (DRG2), a member of the GTP-binding protein superfamily, downregulates SIRT1, leading to enhanced acetylation of p53 and NF-κB p65. In addition, genetic knockout of the DRG2 gene significantly eliminates oxidative stress-induced early senescence [84]. These two studies identified two novel signaling pathways between the SIRT and p53 pathways.

## 2.2.3.3 The role of mTOR pathway in cellular senescence

Oxidative stress activates the PI3K/AKT/mTOR pathway. Under normal circumstances, lipid phosphatase dephosphorylates phosphatidylinositol 3,4,5-trisphosphate (PIP3) to form phosphatidylinositol 4,5-bisphosphate (PIP2), thus terminating the PI3K signaling pathway. In situations of oxidative stress, PI3K catalyzes the phosphorylation of PIP2 into PIP3, initiating the activation of AKT and subsequently triggering downstream signaling pathways, including p53, FOXO, MAPK, mTOR, and NF-κB pathways [85]. Suppression of AKT activation diminishes the generation of superoxide and levels of SOD1 [86]. Disrupted PI3K/AKT signaling initiates diverse molecular pathways, elevating ROS levels through modulation of mitochondrial bioenergetics and induction of nicotinamide adenine dinucleotide phosphate oxidase activation [87]. The mTOR pathway consists of two protein complexes: mTORC1, which activates protein synthesis, lipogenesis, and mitochondrial biogenesis, while inhibiting autophagy to provide energy for combat or escape strategies during stress; and mTORC2 which regulates cell proliferation and cell cytoskeletal rearrangement via protein kinase C signaling through the AKT pathway [88]. Additionally, mTOR upregulates inflammatory senescence-associated secretory phenotype (SASP), including C-X-C motif chemokine ligand (CXCL)-1, interleukin (IL)-6 and IL-8 [53].

In summary, ROS activate PI3K, which in turn activates AKT, leading to further activation of mTOR. Activated mTOR enhances cellular metabolism on one hand and regulates cell proliferation on the other hand. Additionally, mTOR can upregulate the expression of inflammatory factors such as the SASP, the impact of which on cellular senescence will be discussed in the next section (section 2.2.4). This intricate interplay illustrates the complex signaling pathways within cells and their role in the aging process.

## 2.2.4 Glucocorticoids induce a chronic inflammatory response

Senescent cells are capable of releasing a repertoire of signaling molecules recognized as the SASP.SASP includes pro-inflammatory cytokines (such as IL-8, IL-6, IL-1α, and IL-1β), growth factors (such as GM-CSF, TGF-β, and HGF), chemokines (such as CXCL-1/3 and CXCL-10), and matrix remodeling enzymes (such as metalloproteinases) [89]. Continued secretion of glucocorticoid can induce GR desensitization or downregulation, impairing their anti-inflammatory efficacy and thereby amplifying inflammatory reactions [90]. In vitro studies have shown that the emergence of SASP in senescent cells correlates with stimulation of the NF-κB signaling pathway, a key regulator of cellular pro-inflammatory reactions [91]. ROS activate ataxia telangiectasia mutated and inhibit the AMPK pathway, leading to the activation of the NF-κB pathway and promotion of SASP. The NF-κB signaling pathway is the main inducer of SASP under oxidative stress. Excessive ROS activates nucleotide-binding oligomerization domain, leucine rich repeat and pyrin domain-containing protein 3 (NLRP3), which further upregulates the expression of IL-1β and IL-18 by activating caspase-1 [92].

SASP ultimately promotes premature aging and chronic inflammation, and SASP emanating from senescent cells can induce aging in neighboring healthy cells via paracrine signaling [70]. SIPS entails dynamic variations in NF-κB activation, culminating in the secretion of SASP and concurrent augmentation of local STAT3 signaling, which orchestrates the paracrine induction of SASP, thereby hastening cellular aging processes [70] [70]. In summary, chronic excessive release of glucocorticoid exacerbates inflammatory responses, whereby inflammatory factors directly or indirectly impact cellular functionality, accelerating cellular senescence. Additionally, the resultant oxidative stress promotes the release of SASP, which in turn accelerates cellular senescence through paracrine signaling pathways. The detailed mechanism of glucocorticoid-induced cell senescence is shown in Figure 1.

## 2.2.5 Direct impacts of aging on the HPA axis

Finally, aging directly affects the function of the HPA axis. During the aging process, there are changes in the secretion pattern of the adrenal glands, particularly in the quantity and quality of the adrenal cortex, along with a decrease in the sensitivity of the HPA axis to negative feedback of terminal hormones [93]. Cortisol levels tend to increase with age, with a higher average concentration, mainly manifested as elevated nighttime cortisol levels, earlier peak cortisol levels in the morning, decreased cortisol rhythm fluctuations, and a tendency towards irregular secretion patterns [94]. However, there is little change in the secretion of ACTH, which is related to plasma glucocorticoid levels, indicating impaired sensitivity of the HPA axis and reduced negative feedback sensitivity to terminal hormones [95]. Therefore, the dysregulation of cortisol metabolism caused by aging adrenal cells further exacerbates cellular aging.

## 2.3 Sympathetic-adrenal medulla (SAM) axis

In response to stress, the sympathetic nervous system activates, triggering the adrenal medulla to secrete catecholamines such as adrenaline and noradrenaline. Adrenaline is rapidly released within seconds during the stress response, resulting in a 300-fold increase in plasma levels during acute stress [96]. Catecholamines specifically exert their effects by activating G protein-coupled receptors on the cell membrane. Adrenergic receptors (AR), which are members of the G protein-coupled receptors family, consist of two α-ARs and three β-ARs. Catecholamines act on these receptors to increase cellular metabolism [97].

## 2.3.1 Catecholamines promote the production of ROS

In vitro experiments found that catecholamines bind to the cell surface β2-ARs, leading to upregulation of protein kinase A activity, thereby increasing oxidative phosphorylation and generating more ROS [98]. Furthermore, during stress, the activity of the mitochondrial enzyme monoamine oxidase A increases, which also produces more ROS by metabolizing catecholamines and other biogenic amines and generates H_2_O_2_ as a byproduct [65]. Activation of monoamine oxidase A results in the accumulation of impaired mitochondria due to disruption of mitochondrial autophagy, facilitated by parkin inhibition induced by p53, subsequently leading to the activation of CDK inhibitors and initiation of the oxidative stress-induced DDR [65]. Animal experiments found that long-term β-adrenergic stimulation can increase mitochondrial metabolic activity, leading to increased electron flux and elevated intracellular ROS levels [99]. Other animal studies found that prolonged exposure to catecholamines leads to β2-AR activation, increasing ROS production by upregulating nicotinamide adenine dinucleotide phosphate oxidase activity [100], causing oxidative stress, DNA damage, mitochondrial dysfunction, inflammation, and promoting cellular senescence, with mechanisms similar to those activated by the HPA axis. Importantly, in the presence of glucocorticoid, catecholamines exhibit significantly enhanced physiological effects, characterized by increased affinity of catecholamines for β-AR. This is known as the permissive action of glucocorticoid [101]. Therefore, the action of glucocorticoid seems to outweigh the action of catecholamines in SIPS.

## 2.3.2 Catecholamines and cellular senescence

Numerous studies have found that catecholamines induce intracellular oxidative stress and DDR. In vitro studies have found that adrenaline can induce DNA damage in human blood cells primarily through the generation of ROS by adrenaline [102, 103]. Norepinephrine induced DNA damage in three epithelial ovarian cancer cell lines [104] and a human oral keratinocyte cell line [105] possibly mediated by β-AR.

**Figure 2.**
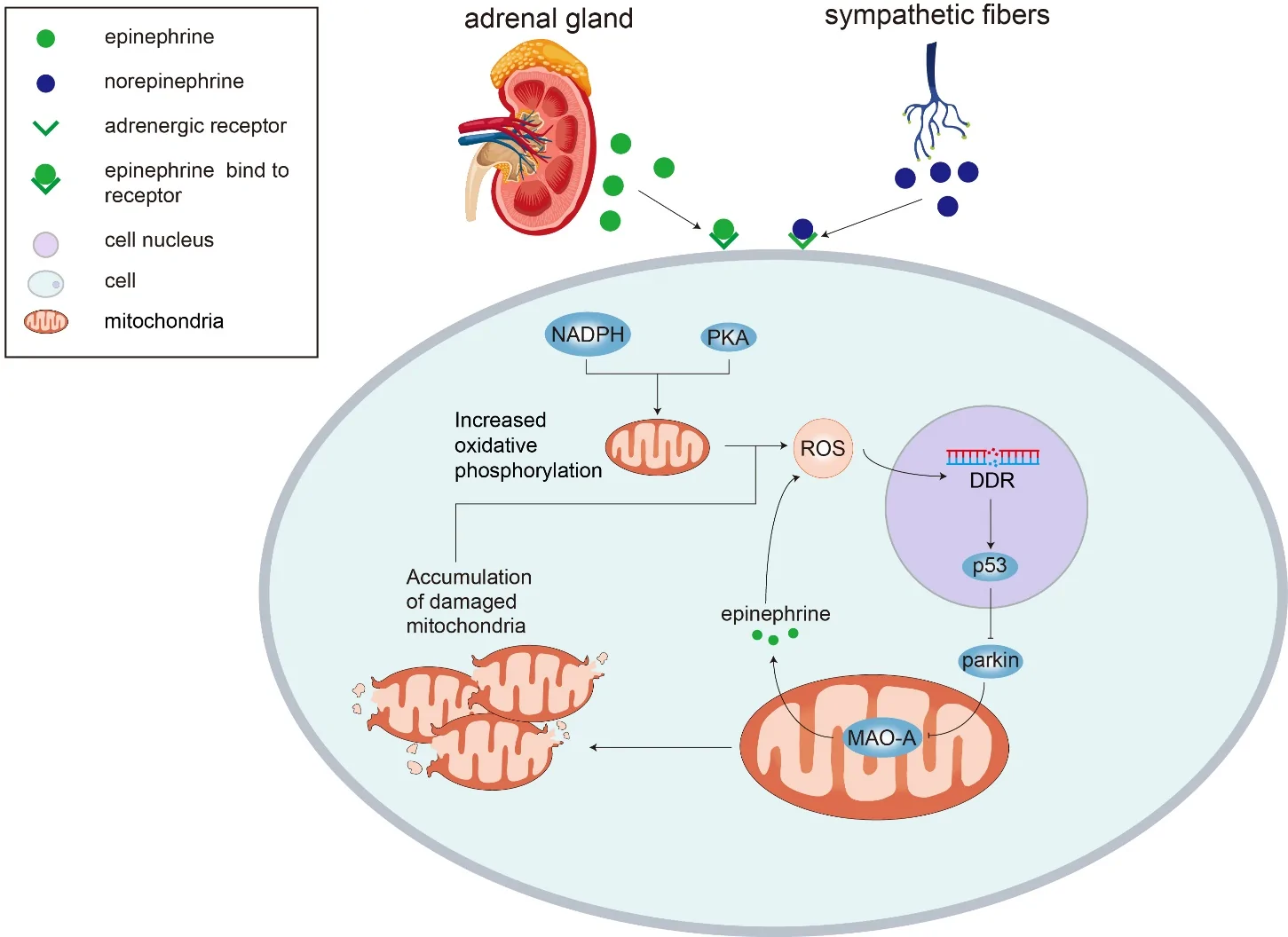
**Mechanisms underlying cellular senescence induced by adrenaline, and catecholamines**. Stress activates the SAM axis, the adrenal glands release adrenaline, and sympathetic nerve endings release norepinephrine,Adrenaline and noradrenaline act on their respective receptors, leading to an increase in mitochondrial electron flux and enhanced ROS production. Additionally, ROS-induced DNA damage response also activates monoamine oxidase A, which degrades adrenaline and generates ROS. Moreover, MAO-A affects mitochondrial autophagy, further promoting ROS accumulation. DDR: DNA damage response; MAO-A: monoamine oxidase A; NADPH: nicotinamide adenine dinucleotide phosphate; PKA: protein kinase A; ROS: reactive oxygen species; SAM: sympathetic-adrenal medulla.

Animal experiments have shown that catecholamine infusion significantly increases catalase activity and decreases SOD activity [106], leading to senescence in mouse endothelial and bone marrow cells, accompanied by increased expression of p53 in cardiac tissue and bone marrow cells [107]. Adrenaline signaling increases the expression of p53 and adhesion molecules in endothelial cells and macrophages [107]. Moreover, the catabolism of catecholamines can precipitate mitochondrial DNA deletions within the adrenal medulla and cortex, a phenomenon exacerbated by aging [108]. Crucially, upon mitochondrial dysfunction, their susceptibility to ROS and stress hormones amplifies, hastening cellular aging in response to subsequent stressors [109]. In addition to β-AR, the action of catecholamines on α-AR also promotes cellular senescence. Li et al. [110] found that norepinephrine acting on α2A-AR increases the expression of β-arrestin 2, a downstream signaling molecule of α2A-AR. β-arrestin 2 promotes the expression of NF-κB, which further activates inflammatory responses, induces the generation of SASP, and promotes cellular senescence. In addition, α2A-AR inhibits cell invasion, migration, and proliferation, while promoting cellular senescence and apoptosis by suppressing the PI3K/AKT/mTOR pathway [111]. The mechanisms by which catecholamines induce cellular senescence are illustrated in Figure 2.

**Table 2:** Important concepts related to SIPS.

	Definition	Relationship with SIPS
**HPA axis**	The HPA axis refers to the Hypothalamic-Pituitary-Adrenal axis, which is responsible for regulating stress response and homeostasis	Activation of the HPA axis results in dysregulated glucocorticoid release, leading to oxidative stress and DNA damage, thereby inducing SIPS [45, 48, 53]
**SAM axis**	SAM axis is Sympathetic Adrenal medulla axis, regulates the acute stress reaction	Following activation of the SAM axis, excessive release of catecholamines induces oxidative stress and DNA damage, leading to the onset of SIPS [65, 98-100]
**Glucocorticoid**	Glucocorticoid is a type of hormone secreted by the adrenal cortex that regulate body metabolism, immune responses, and stress reactions	Prolonged excessive glucocorticoid exposure decreases mitochondrial membrane potential, inhibits mitochondrial biogenesis, impairs ATP production, promotes excessive ROS generation, and ultimately leads to mitochondrial dysfunction. Additionally, excess glucocorticoids can inhibit mitochondrial autophagy, resulting in the accumulation of damaged mitochondria and further production of reactive oxygen species, thereby inducing oxidative stress [29, 30, 35, 40-43]
**Catecholamines**	Catecholamines are neurotransmitters secreted by the adrenal medulla, including adrenaline and noradrenaline, regulating the nervous system and stress responses	Catecholamines activate PKA, promoting oxidative phosphorylation and ROS production. Additionally, catecholamines activate MAO-A, inhibiting mitochondrial autophagy, which leads to the accumulation of damaged mitochondria, further increasing ROS production and promoting oxidative stress [65, 98]
**Mitochondrial dysfunction**	Decreased ability of mitochondria to produce ATP, mitochondrial biogenesis and autophagy are inhibited	Mitochondria are the primary sites of ROS production in cells. Mitochondrial dysfunction, impaired biogenesis, and inhibited autophagy are key factors contributing to the onset of SIPS [29, 30, 35, 40-43]
**Oxidative stress**	Oxidative stress is an imbalance caused by the accumulation of oxygen free radicals within cells, potentially damaging cellular structures and functions	Excessive ROS can impair cellular function, triggering protein and lipid peroxidation, inducing DNA damage, and ultimately leading to irreversible cellular injury and cell death. ROS are primarily regulated by NF-κB and Nrf2 [45, 48, 53]
**Chronic inflammation**	Chronic inflammation is a prolonged and persistent inflammatory response that can lead to tissue damage and disease	Excessive ROS promote the production of SASP, inducing cellular senescence. The SASP released by senescent cells, in turn, induces neighboring healthy cells to undergo senescence [70, 91]
**p53/p21 pathway**	The p53/p21 pathway is a cellular signaling pathway involved in regulating the cell cycle and DNA repair, thereby helping to maintain genomic stability	Upon activation, the p53/p21 pathway inhibits cyclin-CDK complexes, induces Rb dephosphorylation, and promotes Rb-E2F formation, leading to cell cycle arrest at the G1 and S phases. Concurrently, p53 activation also results in mitochondrial dysfunction, contributing to the accumulation of damaged mitochondria [60-62, 64, 65]
**p16 pathway**	The p16 pathway is a cellular signaling pathway primarily involved in inhibiting cell proliferation and helping to maintain normal cell cycle regulation	P16 inhibits the formation of CDK4/6-cyclin D complexes, thereby preventing Rb phosphorylation and slowing down the cell cycle progression [67, 68]
**mTOR pathway**	The mTOR pathway is a cellular signaling pathway that regulates cell growth, metabolism, and survival	Activated mTOR enhances cellular metabolism on one hand and regulates cell proliferation on the other hand. Additionally, mTOR can upregulate the expression of inflammatory factors such as the SASP [53, 88]
**Circadian clock**	It driven by cell-autonomous oscillators, creates 24-hour rhythms in physiology and behavior across various organisms	The circadian clock regulates cellular aging processes through BMAL1, involving oxidative stress pathways (such as Nrf2 and NF-κB), DNA damage response (DDR, like the p53-p21 pathway), and mitochondrial function [115]

BMAL1: brain and muscle arnt-like protein 1; CDK: cyclin-dependent kinase; DDR: DNA damage response; HPA: hypothalamus-pituitary-adrenal; MAO-A: monoamine oxidase; mTOR: mammalian target of rapamycin; NF-κB: nuclear factor kappa-B; Nrf2: nuclear factor-E2-related factor 2; PKA: protein kinase A; Rb: retinoblastoma protein; Rb-E2F: retinoblastoma protein-E2F transcription factor; ROS: reactive oxygen species; SAM: sympathetic-adrenal medulla; SASP: senescence-associated secretory phenotype; SIPS: stress-induced premature senescence

In summary, the mechanism by which catecholamines induce cellular senescence is generally similar to that of glucocorticoid. Although catecholamines have unique pathways to generate excessive ROS in cells, cellular senescence induced by catecholamines appears to be dependent on the presence of glucocorticoid-mediated actions. Up to the present, there are few studies investigating the mechanisms underlying adrenaline-induced cellular senescence, and the specific mechanisms remain unclear, highlighting a gap in current research. Table 2 summarizes key concepts related to the mechanisms of SIPS based on Section 2, aiding in the understanding of SIPS pathogenesis.

## 2.4 Stress hormone and circadian rhythms

The circadian rhythm is an internal timing system found in most organisms and is involved in the feeding-fasting cycle, sleep-wake cycle, activity-rest cycle, and coordination of behaviors and physiology among all organs to adapt to environmental changes [112]. In recent years, the relationship between cellular aging and the circadian rhythm has been increasingly recognized, as these two appear to be closely intertwined. In vitro experiments have shown changes in characteristics of the circadian clock, mainly delayed phase and period extension, in cells undergoing senescence [113]. In animal experiments, Nakamura et al. [114] found that the circadian clock of elderly mice had a longer period, delayed phase, and weakened amplitude compared to young mice. These results suggest that aging can lead to circadian rhythm disruption.

Brain and muscle arnt-like protein 1 (BMAL1) is one of the core genes that regulates the circadian rhythm. BMAL1 forms a heterodimer with circadian locomotor output cycles kaput to regulate the circadian rhythm through a negative-feedback loop and accessory feedback loop [115]. Animal experiments have shown that mice with BMAL1 defects have significantly shortened lifespans and exhibit premature aging phenotypes [116]. BMAL1 deficiency also accelerates the aging phenotype of mesenchymal stem cells in humans and cynomolgus monkeys [117]. Recent studies have found that BMAL1 plays an irreplaceable role in regulating oxidative stress (such as Nrf2 and NF-κB pathways), DDR (such as the p53-p21 pathway), and mitochondrial dysfunction[115]. Therefore, BMAL1 significantly affects the aging process.

It is well-known that the production of cortisol and catecholamines is driven by the circadian rhythm and strictly regulated, with their rhythmicity summarized as lowest at midnight and highest in the morning [118]. Circadian rhythm disruption can lead to dysregulated secretion and persistently high levels of cortisol and catecholamines that accelerate cellular aging. Additionally, cortisol can act on the peripheral clock to regulate the expression of other clock genes, such as transcription factors E4 promoter-binding protein 4, nuclear receptor reverse orientation c-erbA gene β, clock genes Period 2 and transcription factor differentially expressed in chondrocytes 2 [119, 120]. In summary, circadian rhythm disruption caused by changes in clock genes is also an important cause of cellular aging.

## 2.5 The multifactorial nature of cellular senescence

While investigating stress hormones, it is imperative to acknowledge that aging is a complex process regulated by multiple factors. Apart from the direct induction of cellular senescence by stress hormones themselves, factors such as epigenetics, lifestyle and environment also interact with hormones to influence cellular aging. Cellular senescence leads to alterations in epigenetic regulation, manifested by changes in DNA methylation patterns, aberrant post-translational modifications of histones, chromatin remodeling abnormalities, and dysregulation of non-coding RNA functions [121]. Some evidence suggests that epigenetic mechanisms regulate the expression of the GR, thereby influencing the HPA axis and contributing to SIPS [122, 123]. Therefore, epigenetic alterations indirectly contribute to the occurrence of SIPS. Currently, there is limited research on the relationship between SIPS and epigenetics, necessitating further investigation to enhance our understanding of the mechanisms underlying SIPS.

Another lifestyle factor that may contribute to cellular aging is obesity linked to excessive calorie intake, which may lead to abnormal activation of the HPA axis, thereby affecting glucocorticoid secretion. Elevated glucocorticoid levels further promote fat accumulation, forming a vicious cycle. Animal experiments have shown that a high-fat diet can accelerate the aging process [124]. Recent studies suggest that caloric restriction can attenuate cellular aging and caloric restriction aims to reduce calorie intake without malnutrition, thereby extending the lifespan of organisms [125]. A two-year longitudinal cohort study showed that moderate caloric restriction can reduce the production of secreted protein acidic and rich in cysteine, thus potentially extending human lifespan [126]. However, some scholars have proposed that a low-calorie diet (55% to 60% of daily requirements) can induce the body to produce a large amount of heat shock proteins, thereby prompting a stress state and altering the function and activity of mitochondria. The compensatory mechanism produced by heat shock proteins can also shorten lifespan [127]. Currently, there remains some controversy regarding the impact of caloric restriction on aging, and further research is needed to confirm whether it can truly extend human lifespan.

The impact of environmental factors on cellular senescence is increasingly gaining attention in the medical community. Recent research has revealed that ambient inhalable particulate matter, specifically PM2.5, triggers the DDR mechanism and activates the cyclic GMP-AMP synthase-stimulator of interferon genes (cGAS-STING) pathway, thus inducing cellular senescence [128]. Furthermore, in vitro studies have revealed that PM2.5 contributes to cellular aging by downregulating SIRT1 and SIRT3 expression [129, 130]. Clinical studies have identified a correlation between prolonged exposure of females to PM2.5 and shortened telomere length [131]. Beyond air pollutants, water pollution and occupational exposures that can accelerate cellular senescence include exposure to N-nitrosamines, polycyclic aromatic hydrocarbons, lead, pesticides, and hazardous waste compounds and heavy metals, all of which have been found to shorten telomere length [132, 133]. These findings underscore the importance of environmental protection, including reduction of pollutant emissions, and avoidance of occupational exposures in order to prevent premature cellular senescence.

## 3. SIPS and Associated Diseases

Cellular senescence is acknowledged as a key mechanism in the development of numerous chronic diseases. Within these diseases, factors such as oxidative stress, DNA damage, and inflammatory responses lead to aberrant activation and dysregulation of intracellular signaling pathways, resulting in the induction of persistent proliferative arrest in cells. This state leads to the loss of normal cellular function and regenerative capacity. The process of cellular senescence is characterized as a protective response within the cell, aiming to limit the pathological expansion and further dissemination of damaged cells. In this section, we have selected representative diseases to explore their relationship with SIPS.

## 3.1 SIPS and age-related macular degeneration (AMD)

AMD is a chronic progressive disease characterized by an increasing prevalence with age and resulting in central vision decline. It stands as one of the primary etiologies of vision loss globally [134]. The characteristics of AMD include photoreceptor death, as well as degeneration of the retinal pigment epithelium (RPE) and choroidal capillaries [135]. The exact pathogenic mechanism of AMD remains unclear and is likely a result of multifactorial interactions. Among these, the oxidative stress-induced impairment of RPE cells is considered pivotal. An in vitro study demonstrated that oxidative stress-induced premature senescence of ARPE-19 cells manifests characteristics reminiscent of AMD [136]. Accumulation of lipofuscin in the RPE due to oxidative stress-induced damage is believed to trigger cellular injury, RPE dysfunction, and abnormal deposition of extracellular matrix. As RPE dysfunction increases, drusen accumulates, which is a hallmark pathological feature of AMD [137].

The occurrence of AMD is closely linked to the cGAS-STING pathway. Upon recognition of damaged DNA, cGAS catalyzes the production of a secondary messenger called cyclic GMP-AMP by utilizing GTP and ATP. Cyclic GMP-AMP activates STING and impairs the lysosomal clearance of DNA damaged by oxidative stress, further enhancing the STING signaling pathway. This process promotes premature aging of RPE cells through the NF-κB/hypoxia-inducible factor 1 α (HIF-1α) axis, leading to the expression of vascular endothelial growth factor (VEGF) [138-140]. In other words, ROS are also involved in the neovascularization of the retina and choroid. Exogenous ROS provoke the upregulation of VEGF across diverse cell populations, such as vascular smooth muscle cells, endothelial cells and macrophages, via an HIF-1α-mediated pathway [139, 140].

VEGF represents a significant constituent of the SASP in aging RPE cells and senescent RPE cells contribute to dysregulated VEGF levels within the aging retinal microenvironment. Elevated VEGF expression has been noted in senescent cells of various lineages, including fibroblasts, yet its augmentation is not notably correlated with the rise in HIF-1α levels [136]. The activation of the NLRP3 inflammasome in RPE cells plays a key role in the pathogenesis of AMD. Once activated, NLRP3 triggers an inflammatory response through a pathway described earlier. Additionally, the binding of NLRP3 with thioredoxin-interacting protein, which is mediated by ROS, promotes the activation of NLRP3. Thioredoxin-interacting protein modulates AKT-mediated cellular senescence by directly engaging with glucose-induced metabolic stress [141]. In vitro studies have found that markers of cellular aging, such as p16^INK4a^, p21^Cip1^, and p53, are upregulated in damaged RPE cells in AMD [142, 143]. This implies a strong association between the incidence of AMD and SIPS triggered by oxidative stress.

## 3.2 SIPS and chronic kidney disease (CKD)

CKD manifests as a progressive state typified by alterations in both the structure and function of the kidneys stemming from diverse etiologies [144]. In CKD, various stressors such as DNA damage leading to telomere shortening, metabolic stress, oncogenic mutations, inflammatory responses, and mitochondrial dysfunction, can all contribute to cellular senescence. Renal tubular epithelial cells stand out as the predominant cell type linked to renal aging among the diverse cell populations within the kidney [145, 146].

Animal experiments have shown that denervation of the mouse kidney prevents the development of tubulointerstitial fibrosis following unilateral ureteral obstruction and renal ischemia/reperfusion injury [147, 148]. Local infusion of norepinephrine increases the interstitial α-smooth muscle actin, TGF-β1 signaling expression, and excessive deposition of extracellular matrix, leading to a fibrotic response similar to that seen in innervated kidneys [147, 148]. This suggests that sympathetic nerves and norepinephrine promote renal fibrosis. During the period of interstitial fibrosis following ischemia/reperfusion injury, renal tubular cell cycle arrest occurs at the G2/M phase. Renal denervation can alleviate ischemia/reperfusion injury-induced interstitial fibrosis, while norepinephrine injection induces cell cycle arrest in denervated kidneys subjected to ischemia/reperfusion injury [148]. This indicates that stimulation of sympathetic nerves and norepinephrine is a major mechanism triggering renal cell cycle arrest and fibrosis.

Stress hormones also play a crucial role in the progression from acute kidney injury to CKD. Catecholamines promote renal inflammation, affect renal blood flow and glomerular filtration rate, induce renal cell senescence and apoptosis, and promote renal fibrosis, promoting progression of acute kidney injury to CKD [149].

The senescence of renal tubular epithelial cells is chiefly correlated with the upregulation of the wingless-type MMTV integration site family (Wnt)-β-catenin signaling pathway, which regulates cell proliferation and is typically highly conserved. Once activated, the typical Wnt pathway stabilizes β-catenin and translocates it to the cell nucleus. This activates the transcription of downstream target genes that ultimately promoting the expression of genes associated with cell proliferation, survival, differentiation, and migration [150-152]. Animal experiments have fully delineated this mechanism: in CKD, Wnt9a is activated, leading to a significant increase in the expression of fibroblast-specific protein-1 and plasminogen activator inhibitor-1. Both are transcriptional targets of β-catenin. Wnt9a upregulation is significantly associated with the levels of aging-related p16^INK4a^, p19^ARF^, p53, p21, and decreased phosphorylation of Rb [153]. In the rat, Wnt9a stimulation elicits the production of TGF-β1 in aging tubular cells, thereby fostering the proliferation and activation of normal rat kidney fibroblasts. Additionally, the suppression of the AMPK-mTOR signaling axis also contributes to the senescence of renal tubular epithelial cells and podocytes [154].

## 3.3 SIPS and type 2 diabetes (T2D)

T2D is a prevalent chronic metabolic disorder worldwide [155], and stress plays a pivotal role in its’ pathogenesis. The typical characteristics of T2D include impaired insulin secretion and insulin resistance [156] Excessive glucocorticoid exposure fosters the onset of hepatic steatosis, insulin resistance, dyslipidemia, hyper-glycemia, and central adiposity. These metabolic perturbations culminate in the development of T2D, which constitutes one of the most deleterious outcomes of hypercortisolism [157]. Likewise, persistent activation of the SAM axis due to chronic stress increases the circulation of catecholamines. These neurotransmitters foster insulin resistance through the activation of β-ARs [158]. In humans, chronic stress seems to be a hallmark of individuals predisposed to T2D. For instance, individuals with "chronic stress disorders" such as depression exhibit a 60% higher susceptibility to T2D [159]. However, the relationship between SIPS and T2D is currently unclear.

The pathogenesis of T2D is extremely complex, and existing research has demonstrated that cellular senescence is an important contributory mechanism [160]. In T2D, the dysfunction in insulin secretion stems from compromised β-cell function and diminished β-cell mass [161]. In vitro studies have shown that the downregulation of superoxide SOD, catalase, and glutathione peroxidase in β-cells, relative to normal levels, renders them susceptible to the accumulation of ROS induced by elevated glucose and free fatty acid concentrations. Consequently, this heightened ROS burden exacerbates the impairment of β-cell function [162]. Prolonged exposure to high levels of free fatty acids and glucose exerts cytotoxic effects on β-cells, causing endoplasmic reticulum stress, ROS production, or mitochondrial dysfunction, which may lead to dysfunction of telomerase in cells and consequently cause β-cell senescence [163, 164]. At the genetic level, animal studies have found that the expression of p16^INK4a^ in mouse β-cells increases with age, contributing to the decreased regenerative capacity [165]. Moreover, there is an age-associated alteration in the gene expression profile within β-cells, marked by an upregulation of genes associated with cellular senescence, including Cdkn2a and Cdkn2b [160].

In addition to the senescence of pancreatic β-cells, adipose tissue senescence is also an important mechanism leading to T2D. In various organs, the aging of white adipose tissue is called fat aging [166]. Insulin resistance emerges as a consequence of adipose tissue senescence, triggered by the activation of p53. Transgenic mice engineered to overexpress p53 demonstrate accelerated aging and insulin resistance, whereas diabetic mice with p53 inactivation exhibit contrasting effects, mitigating insulin resistance [167, 168]. The transplantation of white adipose tissue from either progeroid or obese mice to control counterparts results in compromised insulin sensitivity and the onset of diabetes. This result underscores the significance of adipose tissue senescence as a pivotal factor in the pathogenesis of diabetes [168]. Crucially, β-cell senescence can be induced and expedited not only by the natural process of aging but also by metabolic stress, notably insulin resistance [161]. This, in turn, leads to impaired β-cell function and promotes the occurrence and progression of T2D.

## 3.4 SIPS and cardiovascular disease (CVD)

Aging significantly impacts the cardiovascular system, precipitating an elevation in CVDs such as hypertension, atherosclerosis, stroke, and myocardial infarction [169]. Studies involving human samples and murine models reveal that aging cardiovascular cells accrue within diseased sites of the cardiovascular system, precipitating conditions such as hypertension, arteriosclerosis and heart failure [170, 171]. Abundant research suggests that endothelial cell senescence is pivotal in the onset of CVD. In vitro studies have identified the presence of aging endothelial cells exhibiting elevated β-galactosidase activity within atherosclerotic plaques in human coronary arteries, thus serving as a hallmark of senescent cell populations [172]. Compared to normal vascular smooth muscle cells, atherosclerotic cells exhibit higher levels of p16^INK4a^, p21^Cip1^, hypophosphorylated Rb, and senescence-associated β-galactosidase activity [173]. Under conditions of stress, in vitro experiments found that H_2_O_2_, tumor necrosis factor alpha, and high glucose all lead to endothelial cell aging. These factors can upregulate the expression of MicroRNA-335-5p, which accelerate endothelial cell aging, while downregulating the anti-aging factor SIRT7, exacerbating endothelial cell oxidative damage and inflammation, and inducing premature endothelial cell senescence [174]. Another in vitro study found that under H_2_O_2_ conditions, LncRNA OIP5-AS1 negatively regulates the expression of miR-4500, thereby upregulating the expression of Arginase 2 and promoting endothelial cell aging. Downregulation of OIP5-AS1 can alleviate endothelial dysfunction in H_2_O_2_-induced aging mice [175].

## 3.5 SIPS and obstructive sleep apnea (OSA)

OSA is a prevalent chronic sleep-related breathing disorder characterized by repeated occurrences of upper airway collapse, resulting in intermittent cessation of breathing [176]. OSA can lead to intermittent hypoxia, triggering oxidative stress and inflammatory reactions, which are primary mechanisms underlying cellular aging. Consequently, OSA may also impact telomere length. However, the relationship between OSA and telomere length remains debated, with some studies suggesting that OSA may result in telomere elongation. In two studies conducted by Polonis et al., a J-shaped relationship was observed between telomere length and the severity of OSA, where individuals with mild OSA exhibited shorter telomeres compared to controls, while those with moderate to severe OSA showed significantly longer telomeres than the control group, independent of age [177, 178]. However, a meta-analysis involving 2639 participants found a significant correlation between OSA and shortened telomere length [179]. In a prospective study, it was demonstrated that OSA leads to a reduction in leukocyte telomere length, with individuals experiencing higher awakening frequencies exhibiting greater leukocyte telomere attrition [180]. Another prospective study found that OSA is linked to an increased prevalence of various aging characteristics in young patients [181]. The hallmark of OSA is recurrent episodes of breathing cessation, leading to intermittent hypoxia, which describes OSA as a form of chronic stress. Studies have indicated that OSA can contribute to an increase in aging markers among patients under the age of 50, including alterations in intercellular communication, mitochondrial dysfunction, and deregulation of nutrient sensing [181]. Oxidative stress and inflammatory responses are pivotal in the pathogenesis of SIPS and telomere shortening attributed to OSA, though not exclusively. Studies have identified that single nucleotide polymorphisms of the Klotho gene significantly mediate the association between OSA and shortened telomeres [182], suggesting that other aging-related genes mediate this process. In animal experiments, exposing mice to a chronic intermittent hypoxia environment resulted in significant telomere shortening in the aorta and renal tissues [183], but the specific mechanism remains incompletely understood. Some scholars have proposed that OSA-induced disruption of circadian rhythms could lead to SIPS and telomere shortening [184], but this currently remains unverified.

In summary, the relationship between OSA and SIPS as well as telomeres is intricate, yielding inconsistent findings across studies. This inconsistency may partly stem from variations in sample selection, differences in study designs, and/or diverse analytical approaches. Further research is required to elucidate the relationship between OSA severity and telomeres shortening, and to clarify, the mechanisms by which OSA induces SIPS and shortened telomeres remain incompletely understood, necessitating more comprehensive animal models to further elucidate these mechanisms.

## 3.6 SIPS and chronic obstructive pulmonary disease (COPD)

COPD is a chronic disease characterized by persistent airflow obstruction and respiratory symptoms, resulting from exposure to inhaled particulate matter such as cigarette smoke and air pollutants, along with developmental, genetic, and social factors [185]. Cellular senescence is recognized as a significant driving mechanism of chronic lung diseases, particularly COPD [186]. Aged cells, including endothelial cells and alveolar epithelial cells, accumulate in the lungs of COPD patients [186]. The oxidative stress induced by exposure to cigarette smoke is likely a significant cause of cellular senescence in COPD patients, as it increases markers of senescence in airway epithelial cells [187]. According to the accelerated aging process in COPD, numerous studies have described an increase in markers of senescence within the lungs of COPD patients. In vitro study has shown that exposing human bronchial epithelial cells to serum from COPD patients significantly increases levels of SA-β-Gal and γ-H2AX [188]. Further research has revealed increased expression of p21 and p16 in small airway fibroblasts of COPD patients, accompanied by mitochondrial dysfunction [189]. Cigarette smoke induces DDR, cellular senescence, and SASP in cultured primary human airway epithelial cells and fibroblasts. It also reduces levels of the shelterin complex protein TPP1 in mouse and human lungs, thereby activating DDR, suggesting that accelerated senescence of airway epithelial cells may occur to some extent through a telomere-dependent mechanism [187, 190]. These findings indicate that COPD-related cellular senescence is also induced by external stressors, leading to DNA damage response (DDR), thereby categorizing this phenomenon as a form of SIPS.

Numerous studies have demonstrated the association between telomere length and COPD. A Mendelian randomization study involving Asian populations found that leukocyte telomere shortening may contribute to increased risk of COPD and interstitial lung disease [191]. In another prospective study following COPD patients for a decade, it was found that over the 10-year observation period, the average telomere length shortened by 183 bp/year in COPD patients. Accelerated telomere shortening was associated with progressive deterioration in lung gas exchange, emphysema severity, and extrapulmonary manifestations in COPD patients. Furthermore, continuous telomere attrition during this period was associated with increased overall mortality risk [192]. Smoking as a risk factor of COPD, and for the length of the telomere is damage. A Mendelian randomization study found that smoking can lead to telomere shortening [193]. Although telomere length in non-COPD smokers tends to be greater than in COPD patients [194]. However, under the influence of smoking, telomere shortening often begins prior to disease onset, accompanied by SIPS. A Mendelian randomization study yielded divergent results, showing no association between telomere shortening and COPD [195]. These results indicate that the relationship between COPD, telomere length, and SIPS remains contentious, necessitating the establishment of multicenter clinical trials for validation.

In summary, the aforementioned diseases are closely associated with SIPS. Therefore, suppressing the onset of SIPS may offer novel insights into the treatment of these diseases. Numerous clinical trials have shown that treating the aforementioned conditions can improve the expression of aging biomarkers and mitigate telomere shortening. Barden et al. found that supplementing n-3 fatty acids in CKD patients significantly increases leukocyte telomere length [196]; Ju et al. observed a significant reduction in the proportion of aged CD8+ cells following treatment with rosuvastatin and ezetimibe in patients with T2D [197]; Ma et al. found a significant increase in leukocyte telomere length following treatment with sitagliptin in patients with T2D [198]; Opstad et al. supplemented selenium and coenzyme Q10 in elderly individuals, finding that supplementation slowed telomere shortening and reduced the risk of cardiovascular mortality [199]; Lin et al. observed an increase in leukocyte telomere length in patients with moderately severe to severe OSA following treatment with oral appliances [200]; Some researchers propose that eliminating senescent cells could represent a novel approach for treating AMD and COPD [56, 201]. The relationship between the above diseases and SIPS is summarized in Table 3. As for therapeutic targets for SIPS, we will discuss them in detail in the next section.

**Table 3:** The role of SIPS in the pathogenesis of relevant diseases.

	Relationship with SIPS
**AMD**	Oxidative stress-induced cellular senescence in RPE cells is a major contributing factor to AMD pathogenesis. This process is closely associated with the cGAS-STING pathway and NF-κB/HIF-1α signaling [137-140]
**CKD**	In CKD, various stressors such as DNA damage leading to telomere shortening, metabolic stress, oncogenic mutations, inflammatory responses, and mitochondrial dysfunction, can all contribute to cellular senescence. Renal tubular epithelial cell senescence is particularly prominent in this context, closely associated with the Wnt-β-catenin signaling pathway [145, 146, 150-152]
**T2D**	Stress promotes the release of glucocorticoids and catecholamines, thereby promoting insulin resistance. Additionally, the aging of pancreatic cells and adipose tissue cells is a key mechanism in the development of T2D [157, 158, 161, 168]
**CVD**	Endothelial cell senescence is one of the important mechanisms contributing to the development of CVD [172-175]
**OSA**	OSA can lead to an increase in markers of aging, closely linked to oxidative stress and inflammatory responses. OSA may affect telomere length, though there is currently debate on this topic [177-181]
**COPD**	Smoking-induced oxidative stress leading to endothelial cell and alveolar cell senescence is recognized as a significant mechanism in the pathogenesis of COPD. There is also controversy regarding whether COPD causes telomere shortening, with some scholars suggesting otherwise [186, 187, 191-195]

AMD: age-related macular degeneration; cGAS-STING: cyclic GMP-AMP synthase- stimulator of interferon genes; CKD: chronic kidney disease; COPD: chronic obstructive pulmonary disease; CVD: cardiovascular disease; HIF-1α: hypoxia-inducible factor 1 α; NF-κB: nuclear factor kappa-B; OSA: obstructive sleep apnea; RPE: retinal pigment epithelium; T2D: type 2 diabetes; Wnt: wingless-type MMTV integration site family

## 4. Therapeutic Targets of SIPS

Aging intervention represents a promising, yet challenging field in medicine, owing to the intricate mechanisms underlying aging, which render anti-aging strategies equally complex. The strong relationship between SIPS and numerous chronic diseases suggests that preventing or slowing, SIPS could benefit individuals who have, or are at risk of, these diseases. In this section, we discuss potential therapeutic targets that could modulate SIPS, as well as the use of existing anti-aging agents used to prevent/slow SIPS.

## 4.1 Nrf2/NF-κB

NF-κB and Nrf2 are pivotal molecules regulating oxidative stress levels within the human body (section 2.2.2). The primary mechanism underlying SIPS involves the excessive generation of ROS leading to oxidative stress. Inhibiting the overactivation of NF-κB or activating Nrf2 could mitigate oxidative stress levels, thereby preventing/slowing SIPS. Animal experiments and in vitro studies have found that activation of Nrf2 significantly inhibits cellular senescence and oxidative stress levels [202, 203]. Other in vitro studies have shown that inhibition of NF-κB can suppress the expression of SASP and aging phenotypes [204].

Quercetin is a flavonoid naturally found in various fruits (such as grapes and peaches) and vegetables (including onions and garlic). It exhibits antioxidative, anti-inflammatory, and mitochondrial function-regulating properties [205]. Shao et al. discovered through in vitro experiments that quercetin significantly inhibits the expression of SASP and aging phenotypes through the Nrf2/NF-κB axis [206]. Moreover, quercetin can also ameliorate SIPS-related diseases such as CVD and T2D by reducing intracellular oxidative stress levels [205]. Another substance, β-cryptoxanthin, is a prevalent carotenoid commonly found in fruits and vegetables like pumpkins, red peppers, and oranges [207]. In vitro studies have found that β-cryptoxanthin preserves mitochondrial function by promoting Nrf2 nuclear translocation, thereby inhibiting SIPS in renal tubular epithelial cells [207]. Recent in vitro studies have discovered that cryptomphalus aspersa egg extract delays aging by promoting DNA damage repair and inducing autophagy [208].

The anti-aging properties of quercetin have been shown in both in vitro and animal studies, and clinical trials investigating quercetin's anti-aging properties are currently underway. By contrast, research on β-cryptoxanthin and cryptomphalus aspersa egg extract is currently limited to in vitro experiments. Further experimentation is needed, but β-cryptoxanthin and cryptomphalus aspersa egg extract could emerge as an effective therapeutic agent for SIPS.

## 4.2 Mitochondria

Mitochondria are the primary site for ROS production. Therefore, actively improving damaged mitochondrial function and eliminating dysfunctional mitochondria could reduce oxidative stress levels. Animal experiments have shown that supplementation with nicotinamide riboside can increase mitochondrial membrane potential in aged mice, improve mitochondrial function, and extend mouse lifespan [209]. Metformin can promote mitochondrial autophagy in diabetic nephropathy mice by activating the PINK1/parkin pathway, thereby alleviating oxidative stress and renal tubulointerstitial fibrosis [210]. Resveratrol improves mitochondrial biogenesis dysfunction through the SIRT1/PGC-1α pathway, reducing excessive ROS production and alleviating oxidative stress [211]. Exogenous supplementation of coenzyme Q10 reduces excessive mitochondrial ROS production and decreases apoptosis of mouse renal cells [212]. Coenzyme Q10 can also promote mitochondrial autophagy, facilitating the clearance of damaged mitochondria [213]. Although these drugs can regulate mitochondrial function and delay the onset of SIPS, more research is needed to establish their safety and efficacy.

## 4.3 Other therapeutic targets

In addition to improves mitochondrial biogenesis dysfunction, resveratrol can induce the expression of SIRT1 while concurrently inhibiting the expression of p53 and p16, exerting an anti-aging effect [214]. FOXO4-DRI competitively inhibits the binding of FOXO4 to p53, inducing cellular apoptosis to attenuate cellular senescence [215]. ABT-263 induces apoptosis in senescent cells by inhibiting BCL-2 [216]. Rapamycin exerts its anti-aging effects through the inhibition of the mTOR pathway [217]. While these drugs alleviate SIPS by clearing senescent cells, they also eliminate the beneficial roles of senescent cells, such as acting as barriers against tumorigenesis. Additionally, drugs like resveratrol and rapamycin can have serious side effects [218]. Therefore, enhancing drug specificity and identifying new therapeutic targets is imperative.

## 5. Conclusion

This article provides an in-depth exploration of how stress hormones induce SIPS, SIPS-related diseases, and potential therapeutic targets for SIPS. Stress, as a lifelong factor, stands as a crucial event leading to SIPS. Stress triggers alterations in the neuroendocrine system, activating the HPA axis and SAM axes, subsequently releasing cortisol, adrenaline, and noradrenaline. Prolonged exposure to these stress hormones disrupts mitochondrial function, leading to the amassment of damaged mitochondria, which subsequently triggers excessive ROS accumulation. ROS can cause DNA damage, oxidative stress, inflammation, and other reactions, thereby inducing cellular senescence. The mechanism of SASP is complex, with current research indicating its close association with various signaling pathways such as the NF-κB, AMPK, mTOR, and p53 pathways. Additionally, SASP is involved in the development of age-related diseases, including AMD, CKD, CVD, T2D, and OSA. Potential therapeutic targets include inhibition of Nrf2, NF-κB and improvement in mitochondrial function. Some drugs such as quercetin, resveratrol, and rapamycin show potential in delaying cellular senescence, but their used requires greater understanding of their safety and efficacy. Future research efforts will focus on improving the specificity and safety of drugs, targeting aspects of aging, aiming to prevent or slow progression of chronic diseases and aging processes. Simultaneously, continued efforts are needed to deepen our understanding of the molecular mechanisms underlying SIPS, by establishing sophisticated models, strengthening interdisciplinary cooperation, and applying new technologies. Advances in our understanding of the cause and prevention of cellular senescence could have marked impacts on human well-being, with secondary benefits to healthcare resource utilization, healthcare costs, productivity, and economies.
